# Ecological Health Assessment of Chinese National Parks Based on Landscape Pattern: A Case Study in Shennongjia National Park

**DOI:** 10.3390/ijerph182111487

**Published:** 2021-10-31

**Authors:** Hang Shu, Chunwang Xiao, Ting Ma, Weiguo Sang

**Affiliations:** 1 Key Laboratory of Ecology and Environment in Minority Areas (Minzu University of China), National Ethnic Affairs Commission of China, Beijing 100081, China; sdshuhang@163.com (H.S.); cwxiao@muc.edu.cn (C.X.); 17400198@muc.edu.cn (T.M.); 2 College of Life and Environment Science, Minzu University of China, Beijing 100081, China; 3 Key Laboratory of Regional Sustainable Development Modeling, Chinese Academy of Sciences, Beijing 100101, China; 4 Institute of Geographic Sciences and Natural Resources Research, Chinese Academy of Sciences, Beijing 100101, China

**Keywords:** landscape pattern, ecosystem health, national park, spatial analysis, VOR model

## Abstract

Assessing the health of the ecosystem based on the landscape pattern of national parks can facilitate policy makers in formulating more targeted conservation policies to better manage national park ecosystems. To analyze the landscape patterns and characteristics of the national park, the ecosystem health evaluation index system of the national park was constructed using the vigor-organization-resilience (VOR) model to evaluate the health status. In this study, the Shennongjia National Park in China was selected as a case study area to be assessed using the index system. The results revealed that the patches of construction land and farmland are the largest in number and the most complex in shape, reflecting the obvious fragmentation of construction land and farmland patches. All patch types in this national park were evenly distributed. The results of the analysis showed that the comprehensive index of national park heath, according to the VOR model, is 0.74, indicating that the ecosystems in this study area were in a good state of health. Ecosystems in strictly protected areas of this park had the highest ecosystem health index levels, while the traditional utilization areas had the lowest. Ecosystem health levels were characterized by significant spatial agglomeration characteristics, with high-high aggregation distribution areas, mainly clustered in strictly protected areas, and low-low aggregation distribution, mainly clustered in traditional utilization areas and marginal areas. This study provided a set of ecosystem health assessment systems and their practical use in China’s newly established national parks.

## 1. Introduction

Maintaining the health of natural ecosystems at a high level can provide adequate and sustainable material support for the development of human society [1]. As a result of the continuous increase in human activity, a series of ecosystem health problems have followed, such as land desertion, habitats loss, greatly reduced forest coverage rate and so on [2]. These problems lead to unhealthy natural ecosystems, seriously hindering sustainable development. In this context, how to protect the health of natural ecosystems to promote sustainable development has not only become the focus of scholars, but has also become a high priority national strategy [3]. Quite a few countries have established various types of protected areas such as national parks to protect significant ecosystems [4,5].

In China, in order to protect the habitat of endangered and precious animals and plants and the integrity of the natural ecosystem, the first nature reserve was established in 1956 [6]. By 2020, China had established more than 11,800 natural protected areas of different levels and types [7]. However, nature reserves have a series of problems such as unclear boundaries, overlapping settings, and prominent contradictions in protection and development [8]. To address these problems, in 2017, China’s government proposed a scheme of setting up a National Park System Pilot Area to better protect natural ecosystems and achieve sustainable development of natural ecosystems [9]. National parks are established to play a vital role in the national nature reserve system. The healthy ecosystem of national parks is conducive to protecting biodiversity and promoting human development [10]. A comprehensive assessment of national park ecosystem health is needed for the construction of high-quality national parks [11]. The aim of this study is to establish a set of methodologies for ecosystem health assessment in national parks in China.

Ecosystem health is considered to be one of the most important issues in ecosystem management, and it is also a key part of ecosystem assessment [12]. A healthy ecosystem should not only be self-active but should also be able to maintain and renew itself [13]. In recent years, there have been lots of related studies on ecosystem health assessment. The ecosystem health assessment involves different scales, which includes global scale, regional scale with administrative units, watershed scales, and specific ecoregional scales [14,15,16,17]. There are also some studies assessing the health status of different ecosystems, such as the aquatic ecosystems of coasts and rivers and the terrestrial ecosystems of agriculture and forests [18,19,20,21]. In terms of the assessment methodology, when considering the scale and characteristics of the study area, it is mainly divided into an indicator species method and an indicator system method. The indicator species method is an indirect method to assess the ecosystem health by monitoring and analyzing the response of representative indicator species to external environmental pressures [22]. The assessing process of this method is relatively complicated. The indicator system method is mainly used to assess the ecosystem health level by establishing the evaluation models [23]. At present, the commonly used evaluation models mainly include the vigor-organization-resilience (VOR) model and pressure-state-response (PSR) model [24]. The VOR assessment model emphasizes the integrity of ecosystem structure and function, including vitality, resilience and landscape pattern within the ecosystem, emphasizing the properties of the ecosystem itself [25]. The PSR assessment model mainly researched causal relationships between ecosystems and human activities, largely considering ecosystem pressure, state, and response [26]. 

As one of the most important types of protected natural areas in China, national parks are subject to the rigorous scientific protection [27]. Therefore, the ecosystem health of national parks is relatively slightly affected by human activities, and the properties of their own ecosystem status are key factors affecting the ecosystems health. At present, most of the ecosystem health studies of various natural protected areas mainly focus on the assessment of the temporal or the process changes of ecosystem health, and there is a lack of ecosystem health studies based on landscape pattern analysis [28]. Consequently, it is necessary to analyze the landscape pattern index to establish a suitable evaluation system to learn the landscape pattern characteristics and the ecosystem health status.

Shennongjia National Park, one of the first batch National Park System Pilot Areas in China, with the most typical evergreen deciduous broadleaf mixed forest ecosystem worldwide. This study area also has a variety of national key protected wild animals and plants such as *Rhinopithecus roxellana*, *Davidia involucrata*, *Taxus wallichiana* var. *chinensis* and so on, which are of global significance for biodiversity conservation [29,30]. It also has great ecological value, so it is of vital significance to protect its ecosystem health [11]. Until now, there have been no studies on ecosystem health assessment based on landscape pattern in Shennongjia National Park. Moreover, this study area has just been established in 2016, and a comprehensive and detailed study of its current ecosystem health is more meaningful for the development of the national park. Therefore, this study takes Shennongjia National Park as a case study and selects VOR model to establish the ecosystem health assessment system based on landscape pattern analysis. We used Fragstats software to explore the landscape pattern of the national park. Based on landscape pattern characteristics, the VOR model was used to study the ecosystem health level, and Arcgis software was used to assess the distribution features of ecosystem health level in Shennongjia National Park in China.

## 2. Materials and Methods

### 2.1. Study Area

**Figure 1:**
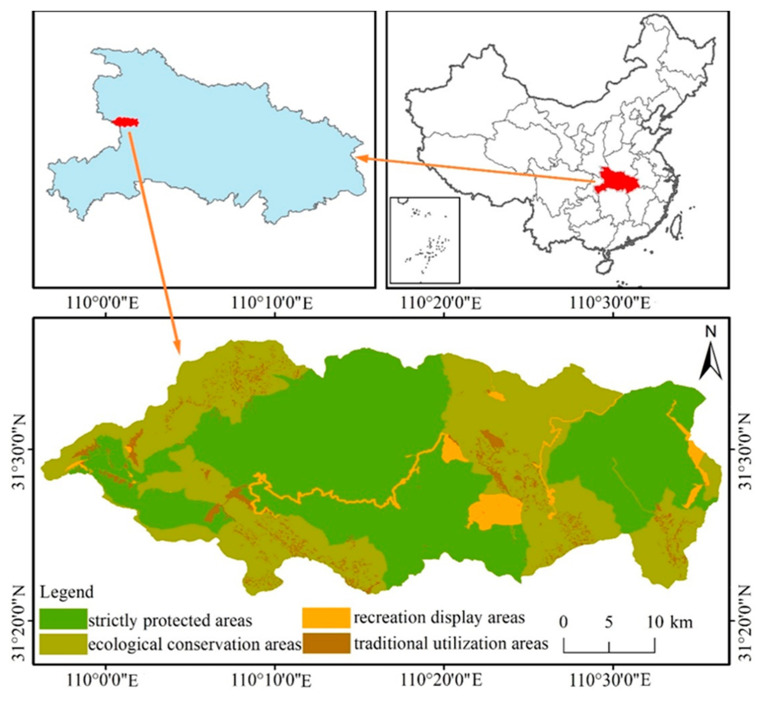
The location of Shennongjia National Park in China and its functional divisions.

Shennongjia National Park is located in Hubei Province of China, with geographic coordinates of 109°56′ E–110°36′ E and 31°17′ N–31°36′ N (Figure 1). It covers a total area of 1167 km^2^. It is located in the northern subtropical monsoon climate zone. The annual precipitation is between 800–2500 mm. Shennongjia National Park has integrated a variety of protected areas in the past, such as national nature reserves, national forest parks and so on [31]. This area is rich in biodiversity and is recognized as one of the key areas for biodiversity conservation in the whole world [32]. According to the characteristics of this national park, there are four main functional divisions in it that have been established, including a strictly protected area, recreation display areas, ecological conservation areas and traditional utilization areas for better performing the natural conservation and socioeconomic functions of national parks [33].

### 2.2. Data Collection and Analysis

#### 2.2.1. Spatial Data Pre-Processing and Classification of Landscape Types

In this study, the Landsat OLI remote sensing images with a resolution of 30 m × 30 m in 2019 was selected as the basic data source. The detailed data parameters are shown in Table 1. These images were obtained from the United States Geological Survey website (http://glovis.usgs.gov/ (accessed on 16 September 2020)). Based on ENVI 5.3 software, the image is interpreted using the supervised classification method of maximum likelihood method. On the basis of supervised classification, combined with forest resource inventory data and Google Earth data, the images were visually corrected to improve the classification accuracy, so as to obtain land use data of Shennongjia National Park. According to the practical requirements, the land use data was classified into ten types: coniferous forest, deciduous broadleaf forest; evergreen broadleaf forest; mixed coniferous broadleaf forest; shrubs; grassland; construction land; farmland; water body and bare land. To ensure a reliable classification, kappa values were used to evaluate the accuracy. The kappa values are larger than 0.850, which meets the application requirements of this study.

#### 2.2.2. Selection of Landscape Pattern Indexes

Firstly, the land use classification map was rasterized and transformed into GRID TIFF format. Then, Fragstats 4.2 software was used to calculate the landscape pattern index of the study area from the class level and landscape level. Although there are many landscape pattern indexes that can be used for landscape pattern analysis, some of them have collinearity [34]. In order to avoid such issues and take regional characteristics into consideration, this study selected corresponding evaluation indexes at the class level and landscape level, respectively, to analyze the landscape pattern of Shennongjia National Park. The ecological significance of the landscape pattern indexes used are shown in Table 2. 

#### 2.2.3. Selection of Grid Size and Landscape Pattern Indexes

According to the ecosystem characteristics of Shennongjia National Park, the VOR model was used to establish the ecological health evaluation indicators. Firstly, the national park ecosystem health assessment was established as the objective layer. Then, according to the objective layer, three indicators of vigor, organization and resilience were established as the principal layer. Finally, nine representative indicator layers are selected from the principal layer. The indicators in the indicator layer constitutes the ecosystem health evaluation index system of national parks (Table 3). 

Acting as one of the most basic functions of the ecosystem, ecosystem vigor can be measured by metabolism or primary productivity [36]. In this study, the vegetation cover was applied to quantify ecosystem vigor, which is an important parameter for describing vegetation growth and ecosystem primary productivity [37]. Based on NDVI, the dimidiate pixel model was employed to calculate the vegetation coverage. The specific calculation method is as follows:

(1) $$Fc=\frac{\mathrm{NDVI}-{\mathrm{NDVI}}_{soil}}{{\mathrm{NDVI}}_{veg}-{\mathrm{NDVI}}_{soil}}$$

where, ${\mathrm{NDVI}}_{soil}$ represents the pixel NDVI value in the area without vegetation cover; ${\mathrm{NDVI}}_{veg}$ represents the pixel NDVI value with complete vegetation cover; Fc represents vegetation coverage, the values of Fc ranged from 0 to 1.

Ecosystem organization is defined as the structural relationships among the components of an ecosystem, which mainly consists of landscape heterogeneity and landscape connectivity [38]. Landscape heterogeneity generally refers to the degree of landscape variation, which can reflect the difference in landscape types. Landscape heterogeneity was measured using Shannon’s Diversity index (SHDI), Shannon’s Evenness index (SHEI) and Patch Richness (PR) [39]. Landscape connectivity was quantified using the Patch Cohesion index (COHESION), Contagion index (CONTAG) and Interspersion Juxtaposition index (IJI) [40].

Ecosystem resilience is the key ability of an ecosystem to stably maintain its structure and function under environmental stresses from nature and society [41]. This study refers to the resilience coefficients of different land use types in previous related studies [12,41,42,43,44]. Different land use types have the specific reference resilience coefficients (Table 4). When calculating the resilience of the ecosystem, the resilience coefficient of coniferous forest, deciduous broadleaf forest, evergreen broadleaf forest, mixed coniferous broadleaf forest, shrubs are classified as resilience coefficient of forest land types. In addition, the richer the biodiversity in an ecosystem, the stronger the ability of the ecosystem to maintain its structure and function. Therefore, the Simpson Diversity index (SIDI) is also used as one of the indicators of ecosystem resilience. 

#### 2.2.4. The Normalization of Evaluation Indicators and the Establishment of Indicator Weights

Normalization of evaluation indicators: When evaluating ecosystem health, the various types of indicators involved are not conducive to unified analysis and evaluation. Therefore, it is necessary to standardize each evaluation indicator and eliminate dimensions to complete the calculation between data [45]. In this study, the range method was used to standardize the data, and the value of the quota layer evaluation indicators were standardized to range from 0 to 1. 

When the increased direction of the single indicator value is the same as the increased direction of ecological health, Formula (2) is used for evaluation. Otherwise, Formula (3) is used for evaluation.

(2) $$P=\frac{R_{\max}-R}{R_{\max}-R_{\min}}$$

(3) $$P=\frac{R-R_{\min}}{R_{\max}-R_{\min}}$$

where, P represents the standardized single indicator value of ecological health; R represents the observed value of a single indicator; $R_{\min}$ represents the minimum value of a single indicator; $R_{\max}$ represents the maximum value of a single indicator.

Weighting of ecosystem health indicators: In the ecosystem health evaluation system, the weight of indicators has an important impact on the evaluation results. The weights indicate the relative importance of the evaluation indicator to the previous one. A reasonable determination of the weight value can improve the accuracy and scientific validity of the evaluation results [46]. The Analytic Hierarchy Process (AHP) method was used in this study. Firstly, the hierarchical structure model which includes objective layer, principal layer and quota layer is constructed by yaahp software. Then, the hierarchical analysis method is used to compare and analyze the importance degree of the indicator under the principal layer (*P*) and the quota layer (*Q*), respectively to construct the judgment matrix. The matrix formula is as follows:

(4) $$P_{k}={\left(Q_{ij}\right)}_{n\times n}=\left[\begin{array}{ccc} Q_{11} & \cdots & Q_{1n}\\ \vdots & \ddots & \vdots \\ Q_{n1} & \cdots & Q_{nn} \end{array}\right]$$

Finally, the constructed judgment matrix will be tested for consistency of the matrix. The formulas are as follows:

(5) $$CM=\frac{CI}{RI}$$

(6) $$CI=\frac{\lambda_{\max}-n}{n-1}$$

where, CM represents the consistency of the matrix; CI represents the consistency indicator; RI represents the random consistent ratio; $\lambda_{\max}$ represents the maximum eigenvalue of the judgment matrix; n represents the order of the matrix.

If the value of CM<0.1, it indicates that the judgment matrix satisfies the consistency. If the judgment matrix cannot satisfy the consistency, the initial value of the judgment matrix needs to be adjusted before performing the consistency check. 

#### 2.2.5. Ecosystem Health Assessment Method and Assessment Grade Determination

According to the weight of each indicator and the standardized single indicator value which was obtained by the above calculation, the ecosystem health value of the national park is obtained by weighted summation. The calculation formula is as follows:

(7) $$E=\sum_{i=1}^{n}S_{i}A_{i}$$

where, E is the national park ecosystem health index of the study area; n is the number of indicators; $S_{i}$ is the standardized single indicator evaluation value; $A_{i}$ is the weight of corresponding indicator. 

This study established the national park ecosystem health assessment criteria by comprehensively considering the actual situation of the national park and the previous evaluation criteria. The national park ecosystem health evaluation standard is divided into 5 grades using an equal-interval approach, which are represented by a continuous real number intervals [0.00, 1.00]. The ecosystem health assessment value approach to 1, the better the ecosystem health is. Conversely, the assessment value approach to 0, the worse the health of the ecosystem is [47]. The following grades were used for the national park ecosystem health index: high-quality health (0.80, 1.00]; good health (0.60, 0.80]; health (0.40, 0.60]; sub-health (0.20, 0.40]; bad health [0.00, 0.20].

#### 2.2.6. Spatial Autocorrelation Analysis

This study is based on the exploratory spatial autocorrelation analysis method to investigate the spatial dependence and agglomeration pattern of ecosystem health level in Shennongjia National Park. Exploratory spatial autocorrelation analysis method includes global spatial correlation and local spatial correlation. It can indicate the degree of interdependence and agglomeration between attributes in a specific area and attributes in other areas. Moran’s *I* index was used to analyze the global spatial agglomeration of the entire study area, Formula (5) is used for calculation [48]. The local Moran’s *I* (LISA) was used to analyze the spatial correlation between the spatial attribute value and its adjacent spatial attribute value, Formula (6) is used for calculation [49]. In the calculation process, permutation tests (999 permutations) were used for evaluating the statistical significance of the Moran’s *I*. Additionally, in order to obtain credible spatial correlation analysis results, the statistical significance value at the 1% level was set.

The Getis-Ord Genral G analysis was used to further explore the characteristics of the agglomeration effects of the ecosystem health level. It can indicate the degree of agglomeration of high and low values. In this calculation process, we set the statistical significance value at the 1% level:

(8) $$\mathrm{Moran}\prime s\;I=\frac{\sum_{i=1}^{n}\sum_{j=1}^{m}w_{ij}(x_{i}-\bar{x})(x_{j}-\bar{x})}{S^{2}\sum_{i=1}^{n}\sum_{j=1}^{m}w_{ij}}$$

(9) $$\mathrm{Local}\;\mathrm{Moran}\prime s\;I=\frac{n(x_{i}-\bar{x})\sum_{j=1}^{m}w_{ij}(x_{j}-\bar{x})}{\sum_{i=1}^{n}{\left(x_{i}-\bar{x}\right)}^{2}}$$

(10) $$G=\frac{\sum_{i=1}^{n}\sum_{j=1}^{m}w_{ij}x_{i}x_{j}}{\sum_{i=1}^{n}\sum_{j=1}^{m}x_{i}x_{j}}$$

where n is the total of spatial units in Shennongjia National Park; m is the number of spatial units geographically adjacent to spatial unit j; i≠j, $S=1/n\overset{n}{\underset{i=1}{\sum }}{\left(x_{i}-\overset{¯}{x}\right)}^{2}$; $x_{i},x_{j}$ represent the ecosystem health value of spatial units i and j; $w_{ij}$ represents the spatial weight matrix of units i and j; $\overset{¯}{x}$ represents the average ecosystem health value. The values of I ranged from −1 to 1, and higher absolute values of I reflect stronger spatial autocorrelations. There is a positive spatial correlation when I>0, a negative spatial correlation when I<0, and no spatial autocorrelation when I=0. There are four types of local autocorrelation: high-high (HH), low-low (LL), high-low (HL), and low-high (LH), which represent the aggregation of spatial units with high ecosystem health levels, the aggregation of spatial units with low ecosystem health levels, and a spatial unit with a high (or low) ecosystem health value surrounded by spatial units with low (or high) ecosystem health values, respectively. 

## 3. Results

### 3.1. Landscape Pattern Characteristics of Class Level

According to the analysis of landscape pattern indexes of CA (total class area), PLAND (percentage of landscape) and LPI, the basic landscape pattern distribution characteristics of different landscape types were obtained (Table 5). Based on the indicators of CA and PLAND, we can obtain the composition of different plaque types in the study area. The indexes of PLAND and the LPI are important bases for determining landscape dominance in the region. It can be found that the main landscape types in the study area are deciduous broadleaf forest, shrubs, coniferous forest and mixed evergreen and deciduous broadleaf forest. These four landscape types account for up to 84.29%, of which deciduous broadleaf forest account for the proportion is the highest at 43.99%. The area of construction land follows the above four types, accounting for 8.46%. The area of grassland, bare land, water body and bare land is very small, and the total area of these four types are merely less than 3%. The order of the PLAND index value and the LPI index value of each landscape type is about the same, while the LPI index value of construction land is relatively large. Combining the index values of the CA, PLAND and LPI, it can be seen that deciduous broadleaf forests, shrubs and coniferous forests occupy a dominant position in Shennongjia National Park, and deciduous broadleaf forests play a major role in maintaining the patch integrity of the national park.

In order to further analyze the landscape fragmentation characteristics of different landscape types in Shennongjia National Park, this study analyzed the indexes of NP, PD and LSI at the class level (Table 3). The largest number of patches (NP) are construction land (676) and farmland (525), accounting for 84.52% of the total number of patches. The number of patches of the above two types of land use is significantly higher than the number of patches of other landscape types. The PD index of construction land (0.58) and cultivated land (0.45) is also significantly higher than other landscape types. From the values of the NP index and PD index, it can be seen that the fragmentation of construction land and cultivated land are obvious. The LSI index of farmland and construction land is relatively high, which shows that the patch shape of farmland and construction land is more complicated. The LSI index value of the bare land and water body is low, which indicates that the patch shape of the bare land and water body is more regular.

### 3.2. Landscape Pattern Characteristics of Landscape Level

Because the ecosystem health assessment is based on the landscape pattern, the indexes of COHESION (Patch Cohesion Index), CONTAG (Contagion Index), IJI (Interspersion Juxtaposition Index), SHEI (Shannon’s Evenness Index) and SHDI (Simpson’s Diversity Index) were selected to analyze Shennongjia National Park’s landscape pattern at landscape level (Table 6). 

The indicators of COHESION, CONTAG and IJI were used to characterize landscape connectivity. The results showed that the index value of COHESION is 99.5035, indicating that the landscape connection degree of the study area is relatively high. The results show that the CONTAG index value is 61.5169, which indicated that the degree of agglomeration of different patch types in the study area belongs to superior middling level. The value of IJI index is 73.0325, which shows that the adjacent sides of the patches are relatively close. These indicators together illustrated that the landscape connectivity of Shennongjia National Park is relatively high. 

The indicators of SHEI and SHDI were usually used to characterize the heterogeneity of the landscape. The SHEI index value of the study area is 0.7066, which indicated that there are unobvious dominant patches in the landscape and the patch distribution is relatively uniform. The SHDI index value of the study area was 1.627, which indicates that the uneven distribution of each patch type in the landscape is not obvious. The SHDI index together with the results of the SHEI index showed that the distribution of patches is relatively uniform. 

### 3.3. Comprehensive Assessment of Ecosystem Health in Shennongjia National Park

Based on the analysis of the landscape pattern of Shennongjia National Park, the size of the scale with 1 km × 1 km grid is used to assess the ecosystem health. According to the indicators and their weights in the VOR model, the ecosystem health of this national park is comprehensively assessed (Table 7).

**Figure 2:**
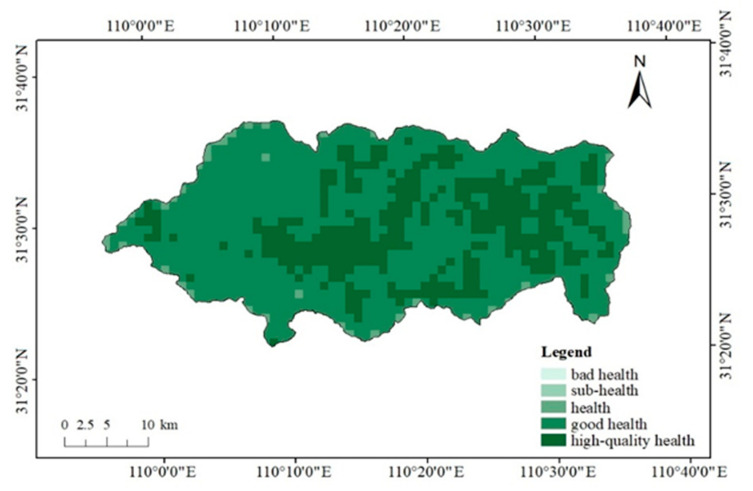
Ecosystem health level in Shennongjia National Park.

The average value of ecosystem health in Shennongjia National Park is 0.74, which indicates that the ecosystem is a good level of health. From the grid level, the ecosystem health of each grid in this study area is at a healthy level and above the healthy level, and 26.38% grids are in high-quality health, 69.05% grids are in good health, 4.57% grids are in a health state (Figure 2).

**Figure 3:**
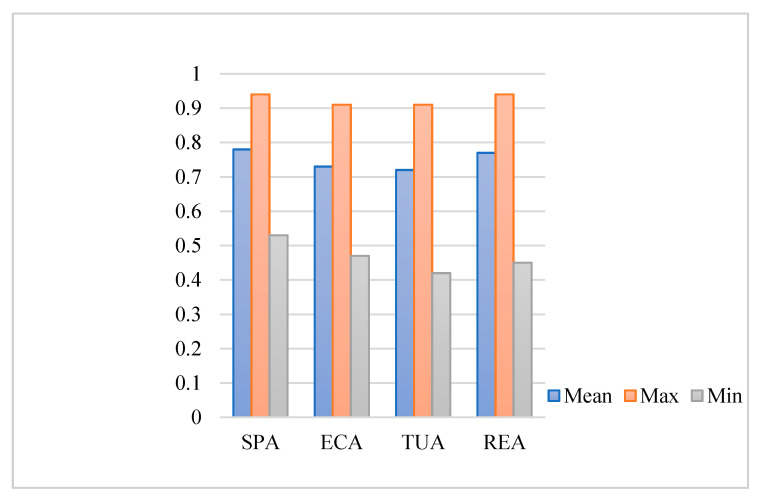
Ecosystem health level in functional divisions of Shennongjia National Park. (SPA, ECA, TUA and RDA are abbreviations of Strictly Protected Areas, Ecological Conservation Areas, Traditional Use Areas and Recreational Exhibition Areas, respectively).

Then, we overlay the distribution map of the ecosystem health level and the functional divisions to get the ecosystem health levels of strictly protected areas (SPA), ecological conservation areas (ECA), traditional use areas (TUA) and recreational exhibition areas (RDA) (Figure 3). SPA have the highest value of the ecosystem health index, with the average score is 0.78, the highest score in the area is 0.94, and the lowest score is 0.53; the ecosystem health index value of the RDA is second, with the average score is 0.77, and the highest score in the area is 0.94, the lowest score is 0.45; the average score of ecosystem health in the ECA is 0.73, the highest score is 0.91, the lowest score is 0.47; the ecosystem health index value of the TUA is the lowest, with the average score is 0.72, the highest score is 0.91, the lowest score is 0.42.

### 3.4. Spatial Pattern Characteristics of Ecosystem Health in Shennongjia National Park

The overall spatial pattern characteristics of the ecosystem health level in Shennongjia National Park are analyzed by the global Moran’s *I* index. The results show that the Moran’s *I* index value is 0.6110 with the z-score 30.08 (>2.58), which was significant at the 1% level. The results implied that the ecosystem health level has a significantly positive spatial autocorrelation in this study area. In summary, the grids with similar ecosystem health levels had remarkable spatial agglomeration effects. Additionally, the analysis results of Getis-Ord General G showed that the observation value of General G is 0.00784 with the z-score 18.25 (>2.58), which was significant at the 1% level. The expected value of General G is 0.00776. The observed value is larger than the expected value. The results indicated that the high-high aggregation distribution is more than the low-low aggregation distribution. 

**Figure 4:**
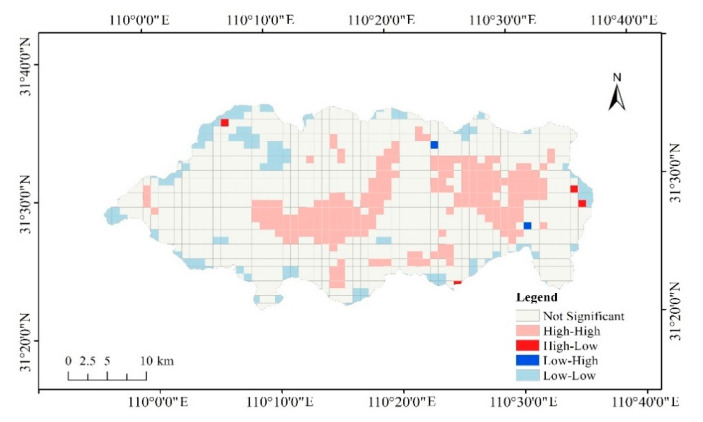
The spatial agglomeration of ecosystem health index in Shennongjia National Park.

The high-high and low-low types of spatial agglomeration of ecosystem health level were analyzed by the local Moran’s *I* index (Figure 4). It can be concluded from the figure that the high-high aggregation distribution areas are mainly concentrated on strictly protected areas of the Shennongjia National Park. The number of grids with high-high type spatial agglomeration accounts for 18.00% of the total number of grids. The low-low aggregation distribution areas are mainly concentrated on traditional utilization areas and edge areas. The number of grids in the low-low type spatial agglomeration accounts for 11.09% of the total number of grids. The number of grids with the insignificant areas account for 70.36%. Through overlay analysis, we found that the high-high type spatial agglomeration areas were mainly concentrated in the strict protection area, while the low-low aggregation distribution areas had no obvious distribution characteristics in the different functional divisions. Additionally, the low-low aggregation distribution areas were mainly distributed in the marginal areas. 

## 4. Discussion

### 4.1. Relationship between Landscape Pattern and Ecosystem Health

Chinese government has established National Park System Plot Area based on its own national natural system conditions. The purpose is to protect the integrity of one or more typical ecosystems and provide places for eco-tourism, scientific research and environmental education, and those designated for special protection, management and utilization Natural area [50]. The establishment of Shennongjia National Park can effectively protect the integrity of the forest ecosystem in the region. 

The number of patches of construction land is the largest, and distribution is relatively scattered. This may be due to the fact that the national park has carried out some infrastructure construction to realize the functions for eco-tourism, scientific research and environmental education in the past [51]. In addition, because of the infrastructure, construction must be built according to actual needs and geographical conditions. This resulted in a relatively scattered distribution of construction land in the national park. The patch shape of the farmland in Shennongjia National Park is the most complex, and the number of patches is followed secondly by the construction land. There are indigenous people who live in the national park. For their livelihood, they need a certain amount of farmland to ensure their daily demands and economic income [52]. Therefore, in order to better protect the ecological system health of the national park, some useful measures should be taken. For example, before building the infrastructure construction, we should make full multi-dimensional assessments of the ecological protection and sustainable development to optimize the quantitative structure and spatial patterns of construction land [53]. In addition, the indigenous people in national parks can increase the diversity of income sources such as planting some under-forest products with the policy permission and using local ecological resources to develop a green economy. In short, the supervisors can make the ecosystem at an optimal health status through reasonable and scientific landscape planning based on the characteristics of the landscape pattern and the results of the ecosystem health evaluation. 

### 4.2. Analysis of Ecosystem Health Status 

The ecosystem health condition of Shennongjia National Park is at a good health level, mainly because, before establishing it, the National Nature Reserve, National Forest Park, World Natural Heritage Site, National Geological Park as well as Dajiuhu National Wetland Park existed in this study area [31]. This means that the ecosystem in this area is well protected and has a high level of ecosystem health. In addition, the vegetation coverage in this study area is high, and the main vegetation type is forest. Among all landscape types studied, forest is the landscape type with the highest resilience index [40]. This is another reason for the high level of ecosystem health in this study area. The ecosystem health level in Shennongjia National Park had a significant spatial aggregation effect, which was related to the high landscape connectivity in the park. In addition, the closer the distance, the more obvious the interregional interaction, which led to the aggregation effect of ecosystem health. This phenomenon is similar to the Bayanbulak World Natural Heritage Site [17]. The high-high aggregation distribution areas are mainly concentrated in strict protected areas, which are often under strict control and protection so that high-high aggregation distribution areas are mainly concentrated here with a high level of ecosystem health. The low-low aggregation distribution was mainly concentrated in the marginal area, which, to a certain extent, reflects the protective effect of the establishment of national parks on the ecosystem. Thus, a certain area outside the marginal area is designated as a buffer zone to improve the health level of the marginal area. 

### 4.3. Implications for Ecological Conservation in Chinese National Park

This research can provide useful suggestions for the development of differentiated ecological protection and restoration measures in the construction of national parks. In regional ecological protection and different areas should take different ecological protection measures [54]. In general, fragile ecological areas should be given priority [55]. Our research has identified regional differences in the ecosystem health level of Shennongjia National Park. The ecosystem health level of traditional utilization areas and marginal areas is relatively low. According to the landscape pattern analysis, the fragmentation degree of construction land and farmland in the traditional utilization areas is high, which may be the main reason for the low level of ecosystem health in this area. Therefore, landscape pattern planning should be strengthened in traditional utilization areas to improve the efficiency of ecological protection. 

Regional ecosystem health assessment is not only important for scientific research, but also has good reference significance for policy formulation and implementation [56,57]. The construction of the Chinese National Park System Plot Area started after 2016. A detailed and comprehensive assessment of the current landscape pattern and ecosystem health status of national parks is of more guiding significance for the development of national parks. The assessment result can also provide reference methods and data for future multi-year ecosystem health assessments of Shennongjia National Park. In addition, the ecosystem health assessment method based on landscape pattern analysis established in this study can also be used to assess other national parks. 

### 4.4. Limitations and Future Research Directions

This study introduces landscape patterns for the first time to assess the ecosystem health of national parks, which can greatly improve the efficiency of national park ecosystem health assessments. However, this study also has certain limitations. 

The weight of each indicator in the VOR model is very important for ecosystem health assessment, and it will directly affect the assessment results [58]. In this study, although the AHP method was used to establish the indicators weight, there were still a certain degree of subjectivity. Therefore, when evaluating the ecosystem health level of national parks, the actual situation of national parks should be fully considered, and corresponding adjustments should be evaluated in multiple directions. Furthermore, the assessment criteria for ecosystem health level with the equal-interval approach has certain deficiencies, which should be based on a large number of practical studies to establish a unified standard. These should be the direction of future efforts. In this study, we evaluated the ecosystem health level of national parks based on the classic VOR model. This model focuses on the characteristics of the ecosystem, but it lacks consideration of the internal biological components of the natural ecosystem and the geographical environment factors. Therefore, the framework still needs to be studied and improved in detail in the future. In addition, we should continue to explore the driving factors and influencing mechanisms of ecological health. These will provide a scientific basis for policy makers and implementors to establish the suitable protection measures. Finally, in future research, we can also explore the relationship between the ecological health of national parks and human health to raise people’s awareness of the protection of national parks, so as to promote the healthy development of national parks. 

## 5. Conclusions

This study highlights the importance of the ecosystem health assessment approach based on the landscape pattern characteristic of exploring the ecosystem health status in Shennongjia National Park. The main research conclusions are as follows: (1) forest land is a dominant landscape type of Shennongjia National Park; (2) the landscape connectivity of the whole study area is relatively high; (3) the ecosystem health level of Shennongjia National Park is at a good health. Additionally, strictly protected areas have the highest level of ecosystem health, while traditional utilization areas have the lowest. (4) The ecosystem health level has a significantly positive spatial autocorrelation in Shennongjia National Park. 

In general, this research analyzed the characteristics of the landscape pattern of Shennongjia National Park, which can provide a guidance for its landscape pattern planning. Through the analysis of the ecological system health level and distribution pattern characteristics of this national park, we have a comprehensive understanding of its ecological health status. The Shennongjia National Park priority reserve proposed in this study can provide a reference to help national park managers formulate more refined protection plans. In addition, this study established one ecosystem health assessment framework of a national park based on a landscape pattern, which can also be applied to other related national parks to improve management efficiency.

## Figures and Tables

**Table 1:** The detailed data parameters of remote sensing images.

Date	Path	Row	Cloudcover	Sunelevation	Sunazimuth
2019/11/03	125	38	2.87	40.36029328	157.45013021
2019/11/10	126	38	5.44	38.36455604	158.26941673

**Table 2:** The ecological significance of the landscape pattern indexes.

Index	Ecological Significance
CA (Total class area)	The CA is the sum of the areas of all the patches in a landscape type.
PLAND (Percentage of landscape)	The PLAND is the total area of a landscape type as a percentage of the total area of the study area.
LPI (Largest patch index)	The LPI is the proportion of the largest patch in a landscape type to the total area of the study area.
NP (Number of patches)	The NP index refers to the total number of patches of a certain landscape type and is usually used to describe the heterogeneity of the entire landscape.
PD (Patch density)	The PD index is an important indicator reflecting the degree of fragmentation and dispersion of the landscape.
LSI (Landscape shape index)	The LSI is an index which can reflect the characteristics of the patch shape.
COHESION (Patch Cohesion Index)	The COHESION index reflects the degree of natural connectivity of related landscape types.
CONTAG (Contagion Index)	The CONTAG describes the degree of agglomeration or spreading trend of different patch types in the landscape.
IJI (Interspersion Juxtaposition Index)	The IJI can reflect the overall interspersion and juxtaposition of various patch types at the landscape level.
SHEI (Shannon’s Evenness Index)	The SHEI is an indicator reflecting the dominant patches and their distribution status in the landscape.
SHDI (Simpson’s Diversity Index)	The SHDI index is sensitive to the uneven distribution of various patch types in the landscape, and it emphasizes the contribution of rare patch types.

Note: Source: Previous research [35]. In this study, the indexes of CA, PLAND, LPI, NP, LSI were used for class level evaluation and the indexes of COHESION, CONTAG, IJI, SHEI, SHDI were used for landscape level evaluation.

**Table 3:** Ecosystem health evaluation indicator system of Shennongjia National Park.

Objective Layer	Principle Layer	Quota Layer
Ecological health assessment of National Park	Vigor	Vegetation Cover Index
	Organization	COHESION (Patch Cohesion Index)
		CONTAG (Contagion Index)
		IJI (Interspersion Juxtaposition Index)
		SHDI (Shannon’s Diversity Index)
		SHEI (Shannon’s Evenness Index)
		PR (Patch Richness)
	Resilience	SIDI (Simpson’s Diversity Index)
		Resilience Index

Note: The landscape pattern indexes in this table are based on the landscape level.

**Table 4:** Ecosystem resilience coefficient of land use type.

Ecosystem Type	Forest Land	Grassland	Construction Land	Farmland	Water Body	Bare Land
Resilience coefficient	0.9	0.8	0.2	0.5	0.8	0.2

**Table 5:** Landscape pattern distribution characteristics of different landscape types.

Landscape Types	CA (ha)	PLAND (%)	LPI (%)	NP (no.)	PD (no./100 ha)	LSI
Coniferous forest	15,094.08	12.9579	5.2406	18	0.0155	8.5720
Deciduous broadleaf forest	51,441.59	43.9896	22.1622	49	0.0421	13.394
Evergreen broadleaf forest	5113.26	4.3896	1.1755	25	0.0215	8.6478
Mixed coniferous broadleaf forest	12,047.49	10.3425	1.4362	25	0.0215	8.0369
Shrubs	19,802.61	17.0000	5.9547	26	0.0223	10.2151
Grassland	2692.98	2.3119	0.3527	51	0.0438	10.9682
Farmland	475.83	0.4085	0.1080	525	0.4507	20.5479
Water body	166.41	0.1429	0.0677	25	0.0215	6.9302
Construction land	9851.22	8.4570	2.6256	676	0.5803	17.9154
Bare land	0.18	0.0002	0.0002	1	0.0009	1.0000

**Table 6:** Landscape pattern characteristics index of the national park.

COHESION	CONTAG	IJI	SHEI	SHDI
99.5035	61.5169	73.0325	0.7066	1.6270

**Table 7:** Comprehensive evaluation of Shennongjia National Park ecological health.

Evaluation Criteria	Evaluation Index	Weight	Standardized Indicator Value	Comprehensive Assessment Value
Vigor (0.279)	Vegetation Cover Index	0.297	0.989	0.74
Organization (0.541)	COHESION (Patch Cohesion Index)	0.116	0.896	
	CONTAG (Contagion Index)	0.116	0.497	
	IJI (Interspersion Juxtaposition Index)	0.039	0.407	
	SHDI (Shannon’s Diversity Index)	0.070	0.399	
	SHEI (Shannon’s Evenness Index)	0.028	0.463	
	PR (Patch Richness)	0.172	0.526	
Resilience (0.163)	SIDI (Simpson’s Diversity Index)	0.027	0.458	
	Resilience Index	0.136	0.937	
